# Supplementary material for: Suppression of Superficial Microglial Activation by Spinal Cord Stimulation Attenuates Neuropathic Pain Following Sciatic Nerve Injury in Rats
Source: Int J Mol Sci. 2020 Mar 30;21(7):2390. doi: 10.3390/ijms21072390 (PMC7177766; doi:10.3390/ijms21072390)
Supplement: Supplementary file 1 [file ijms-21-02390-s001.pdf]

Supplementary

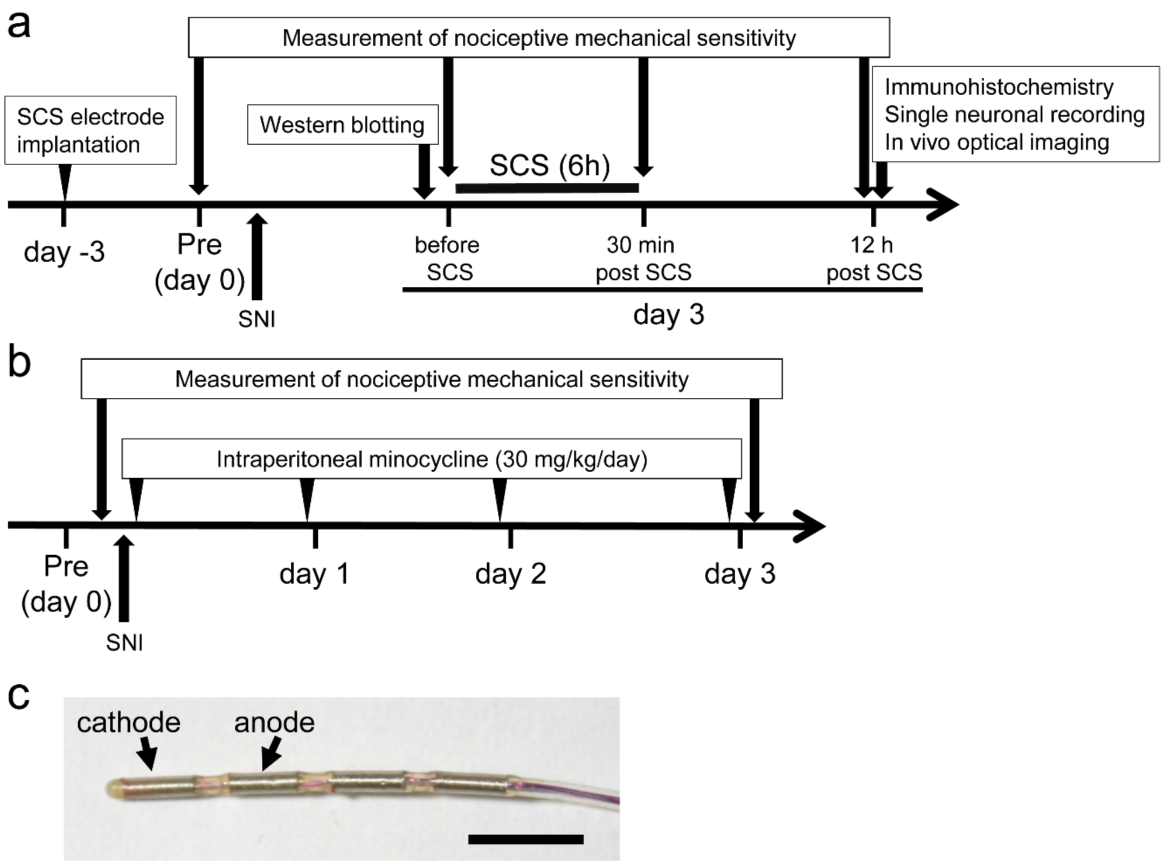

Supplementary Figure S1. Time course of each experiments. Measurement of nociceptive mechanical sensitivity of following SCS (**a**) or following SCS with minocycline (**b**). The implanted electrode (**c**).
